# Atomic PDF analysis as a basis for calculating the electronic structure of quantum materials

**DOI:** 10.1063/4.0000834

**Published:** 2025-10-27

**Authors:** Valeri Petkov

**Affiliations:** Central Michigan University, Mt. Pleasant, MI, USA

## Abstract

Competing interactions in quantum materials lead to the emergence of unusual electronic ground states, including unconventional superconductivity, charge density waves (CDWs), complex magnetic and nematic orders, and Kondo lattices, to name a few. The states are intertwined in intricate phase diagrams and coupled to lattice degrees of freedom that often appear as local structural distortions. There is mounting evidence that the distortions affect the ground states profoundly but are nontrivial to assess precisely using traditional crystallographic techniques, rendering it difficult to understand well why quantum materials behave one way or another.

Recently, atomic pair distribution function (PDF) analysis has proven very useful in studies of materials exhibit structural distortions. However, results from PDF analysis have rarely been used, if at all, as a structural basis for computing and/or predicting their electronic properties by density functional theory (DFT). In the talk, we will demonstrate the advantages of using PDF analysis as a basis for computing electronic properties of quantum materials exhibiting various degrees of structural distortions. Examples will include magnetoelectric FeS, dual heavy fermion APt_2_X_2_ intermetallics (A = U, Ce, or La and X = Si or Ge), NbTe_4_ CDW system (see Fig. 1) and Mott insulator 1-T TaS_2_ [1–4].

## References

[1] V. Petkov and J. Pandey, submitted to Chem. Mater.

[2] V. Petkov, R. Baumbach, M. Jakhar, V. Barone, A. Zafar, L. Gallington, S. Shastri, and B. Aoun, Phys. Rev. B **108**, 224110 (2023). 10.1103/PhysRevB.108.224110

[3] V. Petkov, R. Amin, M. Jakhar, V. Barone, AM Milinda Abeykoon, M. Sretenovic and X. Ke Phys. Rev. B **108**, 174112 (2023). 10.1103/PhysRevB.108.174112

[3] V. Petkov, J. E. Peralta, B. Aoun and Y. Ren, J. Phys. Condens. Matter **34**, 345401 (2022). 10.1088/1361-648X/ac77cf 35688141

